# Transcriptional and Translational Effects of Intronic *CAPN3* Gene Mutations

**DOI:** 10.1002/humu.21320

**Published:** 2010-07-15

**Authors:** Anna Chiara Nascimbeni, Marina Fanin, Elisabetta Tasca, Corrado Angelini

**Affiliations:** 1 Department of Neurosciences, University of PadovaItaly; 2 Venetian Institute of Molecular MedicinePadova, Italy

**Keywords:** *CAPN3*, LGMD2 A, calpainopathy, intronic variants, pathogenetic mutations, splicing

## Abstract

Variants of unknown significance in the *CAPN3* gene constitute a significant challenge for genetic counselling. Despite the frequency of intronic nucleotide changes in this gene (15–25% of all mutations), so far their pathogenicity has only been inferred by *in-silico* analysis, and occasionally, proven by RNA analysis. In this study, 5 different intronic variants (one novel) that bioinformatic tools predicted would affect RNA splicing, underwent comprehensive studies which were designed to prove they are disease-causing. Muscle mRNA from 15 calpainopathy patients was analyzed by RT-PCR and splicing-specific-PCR tests. We established the previously unrecognized pathogenicity of these mutations, which caused aberrant splicing, most frequently by the activation of cryptic splicing sites or, occasionally, by exon skipping. The absence or severe reduction of protein demonstrated their deleterious effect at translational level. We concluded that bioinformatic tools are valuable to suggest the potential effects of intronic variants; however, the experimental demonstration of the pathogenicity is not always easy to do even when using RNA analysis (low abundance, degradation mechanisms), and it might not be successful unless splicing-specific-PCR tests are used. A comprehensive approach is therefore recommended to identify and describe unclassified variants in order to offer essential data for basic and clinical geneticists. ©2010 Wiley-Liss, Inc.

## INTRODUCTION

Autosomal recessive limb girdle muscular dystrophies (LGMD) are a group of disorders characterized by progressive involvement of proximal limb girdle muscles, including at least 14 different genetic entities. LGMD2A [MIM# 253600] is the most prevalent form of LGMD in many countries, and is caused by mutations in the *CAPN3* gene [MIM# 114240] encoding *calpain-3* protein. About 350 different *CAPN3* gene mutations have so far been reported on the Leiden Muscular Dystrophy Database, most of which are private and distributed throughout the gene. Approximately 70% of mutant alleles are of missense type; the remainder are null mutations (deletion/insertion causing frame shifting, nonsense, and splice site mutations), large genomic rearrangements, and synonymous or intronic changes causing aberrant splicing.

While the truncating mutations are considered to be causative, the pathological significance of nucleotide changes localized in intronic regions is very difficult to predict, which makes distinguishing between benign and pathogenetic variants a challenge and compromises a conclusive genetic counselling. Indeed, many intronic changes remain of *uncertain clinical significance (UVs)*, thus partially explaining the observation that, although a severe calpain-3 protein defect in muscle usually corresponds to primary LGMD2A, about 20% of biochemical defects remain without any molecular proof [Fanin et al., 2009a]. Many *CAPN3* intronic variants have been identified during diagnostic screening: they account for about 15% of the total variants listed at the Leiden Database (28% in splice sites, 72% in deep intronic position), and for about 25% of the mutations reported in other studies [Blazquez et al., 2008; Krahn et al., 2006]. However, it is conceivable that the true frequency of intronic mutations has largely been underestimated because deep intronic sequences are not conventionally sequenced and, most aberrant transcripts are expressed at low abundance and are usually prone to degradation by nonsense-mediated mRNA decay (NMD) mechanism [Maquat, 2004]. For the majority of intronic variants the consequences on mRNA splicing have been only inferred by *in-silico* analysis [Duno et al., 2008], whereas experimental demonstration of their pathogenicity has been obtained by mRNA studies for only 1% of them [Leiden Database; Haffner et al., 1998; Krahn et al., 2007; Blazquez et al., 2008]. In this study we have exploited the availability of diagnostic muscle biopsies to demonstrate previously unrecognized pathogenetic effects, at both transcriptional and translational levels, of 5 different intronic mutations in the *CAPN3* gene.

## MATERIALS AND METHODS

### Selection of patients and muscle biopsies

From a population of over 100 LGMD2A patients who had received a biochemical and genetic characterization in our Centre, 16 cases were selected because of the presence of one intronic variant in the *CAPN3* gene with an unknown pathogenetic effect. In all cases except one, the second mutant allele has been identified. Patients underwent diagnostic open muscle biopsies, after written consent had been obtained. As normal controls, we used genomic DNA, cDNA from muscle tissue and calpain-3 protein from subjects who had resulted free of any neuromuscular disorders.

### Protein analysis by western blotting

Semi-quantitative analysis of calpain-3 protein in muscle was conducted as reported [Fanin et al. 2009b]. The quantity of immunoreactive bands at 94 kDa (full-length protein) was determined by densitometry and expressed as a percentage of control.

### In-silico analysis of unclassified intronic variants (UVs)

Prediction of the potential effect of intronic variants was determined by the following splice-site prediction programs (SSPPs): Human Splicing Finder (HSF V2.4 at http://www.umd.be/HSF) [Desmet et al., 2009], Splice Site prediction by Neural Network (NNSPLICE V0.9 *at* http://www.fruitfly.org/seq_tools/splice.html), Splice View (http://bioinfo.itb.cnr.it/oriel/splice-view.html) and NetGene2 (*at* http://www.cbs.dtu.dk/services/NetGene2). Score variation in canonical splice-site use and potential activation of cryptic splice-sites in the presence of mutations were evaluated by analysis of exon and intron sequences localized in proximity to mutations using default settings (Supp. Table S1).

### RNA analysis

Total RNA was isolated from muscle biopsies using the SV Total RNA Isolation System kit (Promega, Madison, WI) including treatment with DNAsel, and reverse transcribed to cDNA with Superscript III reverse transcriptase (Invitrogen, San Diego, CA) and random hexamers. In one patient the total RNA was isolated also from blood using the PAXGene blood RNA System (PreAnalytiX, Qiagen, Hilden, Germany). cDNA was amplified with specific calpain-3 primers designed on the published human calpain-3 mRNA sequence (GenBank accession number: NM_000070). PCR products were analyzed by gel electrophoresis: aberrant bands were manually excised and processed using the QIAquick Gel Extraction kit (Qiagen) for sequencing. Furthermore, we designed splicing-specific and skipping-specific primers (sequence available on request) to selectively amplify abnormal transcripts predicted by bioinformatic tools, even when this was not detectable on RT-PCR. Each splicing-specific PCR was tested on both patients and normal controls and directly sequenced. Details of *in-silico* predictions of splicing are reported in Supp. Table S1.

## RESULTS

We investigated the pathogenetic effect in muscle of 5 different intronic variants (one novel) in the *CAPN3* gene, including 3 nucleotide substitutions in the canonical donor or acceptor splice sites and 2 more deep intronic variations, and demonstrated that they all caused abnormal mRNA splicing and abolished or impaired protein synthesis (Table 1, Table 2, Supp. Table S1). These intronic mutations accounted for 9% of the total mutant alleles and 7% of all the mutations found in our population of over 100 LGMD2A patients.

**Table 1:** Consequences of Intronic Mutations at RNA Level

Intronic sequence variant*	Alternative acceptor/donor site, position	Effect at RNA level		Theoretical effect at protein level*	Methods	Interpretation
**c.1030-1G>A**	**A1/ c.1030-1**	r.1030delG	deletion of the first bp exon 8	p.V344SfsX8	cDNA amplification, sequencing	pathogenetic

**c.1524+1G>C**	**D1/c.1426**	r.1425_1524del	deletion of 99 bp exon 11	p.V476_E508del	cDNA amplification, gel extraction of aberrant band, sequencing	pathogenetic
	
	**D2/c.1524+213**	r.1524_1525ins1524+1_1 524+31;1524+1g>c	exonization of 212 bp intron 11	p.E508 V509ins 508+1_508+70	Splicing specific-PCR, sequencing	pathogenetic

**c.1992+1G>T**	**WT D/c. 1992+1**	r.1915_1992del	skipping of exon 17	p.P639_D664del	Splicing specific-PCR, sequencing	pathogenetic
	
	**D1/c.1992+32**	r.1992_1993ins1992+1_1 992+31;1992+6g>u	exonization of 31 bp intron 17	p.D664_D665ins 664+1_664+10	Splicing specific-PCR, sequencing	pathogenetic

**c.1193+6T>A**	**D1/c.1193+32**	r.1193_1194ins1 193+1_1 193+31;1193+6u>a	exonization of 31 bp intron 9	p.M399X	cDNA amplification, gel extraction of aberrant band, sequencing	pathogenetic

	**A1/c.1746-21**	r.1745_1746ins1746-19_1746-1;1746-20c>g	exonization of 19 bp intron 13	p.E582 E583ins 582+1_582+6fsX9	Splicing specific-PCR, gel extraction of aberrant band, sequencing	pathogenetic
	
	**A2/c.1746-88**	r.1745_1746ins1746-86_1746-1;1746-20c>g	exonization of 86 bp intron 13	p.E582 E583ins582+1_582+28fsX41	Splicing specific-PCR, gel extraction of aberrant band, sequencing	pathogenetic
	
**c.1746-20C>G**	**A3/c.1746-124**	r.1745_1746ins1746-122_1746-1;1746-20c>g	exonization of 122 bp intron 13	p.E582 E583ins 582+1_582+30fsX53	Splicing specific-PCR, gel extraction of aberrant band, sequencing	pathogenetic
	
	**A4/c.1746-307**	r.1745_1746ins1746-305_1746-1;1746-20c>g	exonization of 305 bp intron 13	p.E583X	Splicing specific-PCR, sequencing	pathogenetic
	
	**WT A13/c.1537-2**	r.1745_1746insl745+1_1 746-1;1746-20c>g	exonization of entire intron 13	p.E583X	Splicing specific-PCR, gel extraction of aberrant band, sequencing	non pathogenetic

WT: Wild type; D: donor site; D1, D2, etc: alternative cryptic donor sites; A: acceptor site; A1, A2, etc: alternative cryptic acceptor sites; exonization: intron retention

* Human Genetic Variation Society (HGVS) approved guidelines (http://www.hgvs.org/mutnomen). GenBank accession number AF209502.1.

**Table 2:** Consequences of Intronic Mutations at Protein Level

Intronic sequence variant*	Case N.	Second mutant allele	Protein amount (% of control)
**c.1030-1G>A**	7161	c.550delA, p.T184RfsX36	0

**c.1524+1G>C**	7555	c.755T>C, p.M252T	5

	1324	c.1193+6T>A	0
	
**c.1992+1G>T**	1754	c.1061T>G,p.V354G	0
	
	5393	c.1343G>A, p.R448H	0

	1324	c.1992+1G>T	0
	
	3393	c.1468C>T, p.R490W	100§
	
**c.1193+6T>A**	5427	not identified	20
	
	6804	c.309+4469_1116-1204del, p.E104MfsX11	20
	
	7652	c.1303G>A,p.E435K	50
	
	8242	c. 1469G>A, p.R490Q	100§

	1338	c.1333G>A,p.G445R	0
	
	2522	c.245C>T, p.P82L	20
	
**c.1746-20C>G**	4622	c.1061T>G,p.V354G	10
	
	6211	c.610C>G, p.L204V	0
	
	6385	c.550delA, p.T184RfsX36	5
	
	7894	c.697G>C, p.G233R	10

* Human Genetic Variation Society (HGVS) approved guidelines (http://www.hgvs.org/mutnomen). GenBank accession number AF209502.1.

§ protein with loss of function (autolytic activity).

### Variation c.1030-1G>A (intron 7)

This was the first time this mutation had been identified in an LGMD2A patient. The patient was a compound heterozygote for a null mutant allele (c.550delA). This mutation abolishes the canonical acceptor splice-site, but the expected skipping of exon 8 was not detected. From SSPPs analysis, this intronic variant was expected to create a novel acceptor splice site (A1) with consequent deletion of the first nucleotide of exon 8 (r.1030delG). cDNA sequencing confirmed this deletion, which resulted in the creation of a downstream premature stop codon (p.V344SfsX8). Calpain-3 protein in muscle was absent, demonstrating the deleterious effect of this mutation at translational level.

### Variation c.1524+1G>C (intron 11)

**Figure 1:**
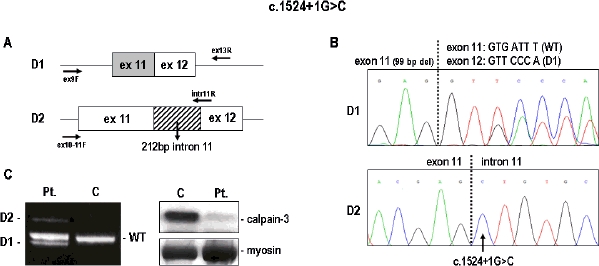
Splicing analysis of the c. 1524+1 G>C variant. **Panel A.** Schematic representation of the splicing patterns generated by this variant: D1, carrying the deletion of the last 99bp of exon 11 (gray box), and D2, with the retention of 212bp of intron 11 (dashed box), resulting from the use of the alternative donor splice sites D1 and D2, respectively. The arrows indicate the localization of the primers used. **Panel B.** Sequences from PCR amplification of muscle cDNA from a heterozygous mutant patient, showing the co-amplification of the wild-type (WT) and the alternately spliced mRNA (D1), and from splicing-specific PCR amplification, which selectively amplifies the allele carrying the mutation (D2) in the same patient. **Panel C.** RT-PCR analysis of normal control (C) and a heterozygous mutant patient (Pt.) who shows the WT product and two additional low-abundant products corresponding to the alternately spliced mRNA (D1 and D2). Calpain-3 western blot shows that this mutation caused a severe reduction of protein (Pt.) corresponding to about 5% of control (C) after myosin normalization.

This mutation was identified in one LGMD2A patient in our series, who was a compound heterozygote for a missense mutant allele (p.M252T) and showed severely reduced calpain-3 protein level in muscle (5% of control), revealing its deleterious effects at translational level. This mutation abolishes the canonical donor splice-site, but a transcript carrying the skipping of exon 11 was not detected, even when using skipping-specific primers. Following SSPPs analysis, this variant was expected to produce two different aberrant transcripts (Figure 1): one originated by the use of a cryptic donor site (D1), leading to a deletion of the last 99 basepairs in exon 11 (r. 1425_1524del), and another (D2) resulting from the exonization (intron retention) of 212 basepairs in intron 11 (r.1524_1525ins1524+1_1524+31;1524+1g>c; p.E508_V509ins 508+1_508+70). D1 transcript was characterized by cDNA amplification, which showed a shortened product corresponding to the expected deletion, that was identified by sequencing (p.V476_E508del). A further confirmation was obtained by PCR with D1 splicing-specific primers. While this transcript was sufficiently expressed and stable enough to be easily detected by cDNA analysis in muscle, the D2 transcript was detected only by splicing-specific PCR.

### Variation c.1992+1G>T (intron 17)

**Figure 2:**
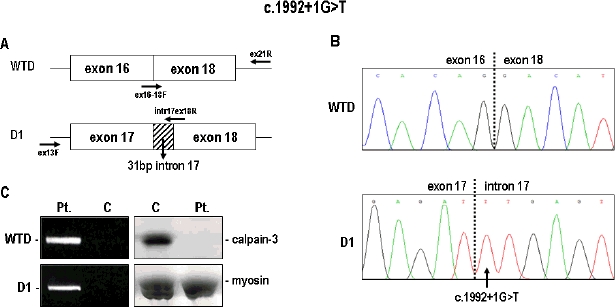
Splicing analysis of the c. 1992+1 G>T variant. **Panel A.** Schematic representation of the two splicing patterns generated by this variant: WTD, carrying the skipping of exon 17, and D1, with the retention of 31 bp of intron 17 (dashed box), resulting from the use of the alternative donor splice site D1. The arrows indicate the localization of the primers used. **Panel B.** Sequences from splicing-specific PCR amplification of muscle cDNA from a heterozygous mutant patient, showing the two aberrant transcripts (WTD and D1) generated by this variant. **Panel C.** Splicing-specific PCR amplification showing the selective amplification of the aberrant transcripts generated by this variant (WTD and D1) only in the heterozygous mutant patient (Pt.). Calpain-3 western blot in a patient (Pt.) shows that this mutation produced absent protein.

This mutation was identified in 3 unrelated LGMD2A patients in our series; its deleterious effect at translational level was demonstrated by the observation that it was associated with absent or virtually absent protein when found in a compound heterozygote state with different mutant alleles. The mutation abolishes the canonical donor splice-site and we demonstrated that it caused the skipping of the exon 17 (r.1915_1992del; p.P639_D664del) in muscle. This result was confirmed also in blood mRNA from one patient. The resulting transcript (WTD) is probably unstable and poorly expressed, since it was detectable only with skipping-specific primers. At least 3 cryptic splicing sites in the region surrounding this mutation obtained a high score from SSPPs analysis, but only one of them was shown to be effectively activated (D1) in muscle and blood cDNA by splicing-specific primers. This cryptic splice site generated an aberrant transcript carrying the insertion of 31 basepairs in intron 17 (r.1992_1993ins1992+1_1992+31;1992+6g>u; p.D664_D665ins 664+1_664+10) (Figure 2).

### Variation c.1193+6T>A (intron 9)

**Figure 3:**
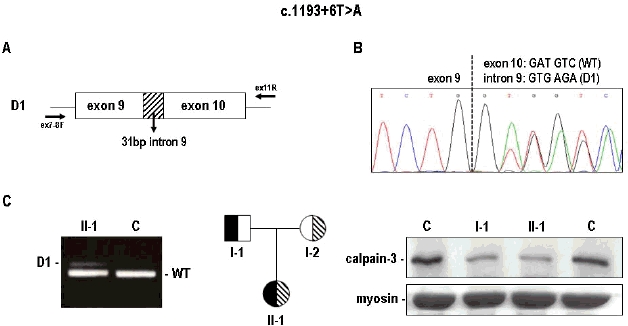
Splicing analysis and translational effect of the c. 1193+6 T>A variant. Panel A. Schematic representation of the aberrant splicing product (D1) generated by this variant, carrying the insertion of 31bp of intron 9 (dashed box) and resulting from the use of the alternative donor splice site D1. The arrows indicate the localization of the primers used. Panel B. Sequence from PCR amplification of muscle cDNA from a heterozygous mutant patient, showing the co-amplification of the WT and the alternately spliced mRNA (D1). Panel C. RT-PCR analysis of normal control (C) and a heterozygous mutant patient (II-1) who shows the WT product and one additional low-abundance product corresponding to the alternately spliced mRNA (D1). Family pedigree (case n. 7652) and western blot show that this intronic mutation (filled symbol) produced a reduction of calpain-3 protein of about 50% of control (C) after myosin normalization, as assessed in the muscle biopsy from both the heterozygous father (I-1) and his affected daughter (II-1), who was a compound heterozygote for a second missense mutation (p.E435K, dashed symbol).

This mutation was identified in 6 unrelated LGMD2A patients in our series, all from the same administrative district of the Veneto Region (a possible founder effect followed by genetic isolation might have occurred). Among the 6 patients with this mutation, one was the object of a family study (Figure 3): we conducted both a segregation analysis of the mutant allele and a study of the effect of this mutation at calpain-3 protein level in 2 different family members (a muscle biopsy was obtained from both an affected girl and her heterozygote father, who reported hyperCKemia before the diagnosis was obtained in his daughter).

The deleterious effect of this mutation at translational level was demonstrated by the observation that: 1) when it was associated with a null mutant allele, it produced absent or very reduced amounts of protein. This variant was not predicted to cause the loss of the canonical donor splice site, but the score for its use was reduced by all the SSPPs algorithms used. This means that the generation of a correctly spliced transcript would still be possible *in-vivo*, but our protein data suggest that, if this is the case, this might only take place to a very limited extent; 2) when it was expressed in heterozygote state, it produced one half of the amount of protein; 3) when associated with a missense mutant allele, which, as we had previously reported, caused the loss of functional autolytic activity without any quantitative defect, it produced normal protein quantities. Furthermore, following SSPPs analysis, this variation was expected to affect correct splicing by use of an alternative cryptic donor splice site (D1), resulting in the insertion of 31 basepairs at the beginning of intron 9 (r. 1193_1 194ins1 193+1_1193+31; 1193+6u>a; p.M399X). The D1 transcript was sufficiently expressed to be detected by RT-PCR analysis: it produced an extra-band which was detectable after gel electrophoresis and which was excised and sequenced (Figure 3). No previous functional characterization of this mutation has been provided.

### Variation c.1746-20C>G (intron 13)

This mutation was identified in 6 unrelated LGMD2A patients in our series, and also in several previous reports, where its pathogenetic effects have been suggested [Piluso et al., 2005; Hermanova et al., 2006; Leiden Database], but unsupported by experimental data [Stehlikova et al., 2007; Krahn et al., 2007], or even reported as a polymorphism [Groen et al., 2007; Leiden Database].

**Figure 4:**
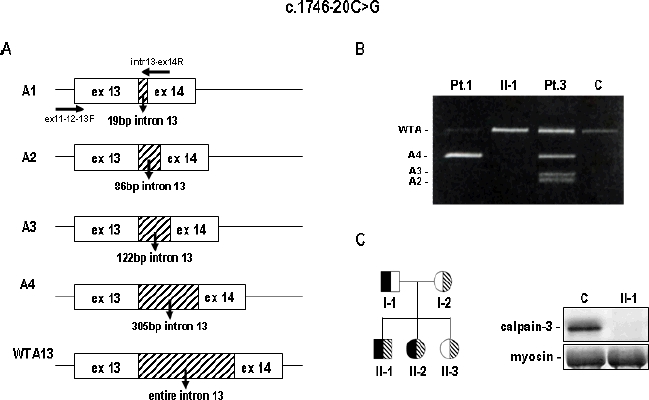
Splicing analysis and translational effect of the c. 1746-20 C>G variant. **Panel A.** Schematic representation of the 5 splicing patterns generated by this variant, resulting from the use of the cryptic acceptor splice sites A1, A2, A3, A4, carrying the retention of 19, 86, 122 and 305 bp of intron 13 (dashed boxes), respectively, and WTA13, with the retention of the entire intron 13 (dashed box). The arrows indicate the localization of the primers used. **Panel B.** Splicing-specific PCR (designed to potentially amplify all the 5 aberrant transcripts) showing that these transcripts are variably expressed and not always detectable in the heterozygous mutant patients (Pt. 1, II-1, and Pt.3). The WTA transcript is expressed also in normal control (C). **Panel C.** Family pedigree (case n. 6211) and western blot showing that this intronic mutation (filled symbol) produced a deleterious effect at protein level, as demonstrated by the complete loss of calpain-3 protein in the muscle from one affected patient (II-1) who was a compound heterozygote for a second missense mutation that has a deleterious effect as well (p.L204V, dashed symbol).

Conversely, in our study, the deleterious effect of this mutation has definitely been demonstrated at both translational (when this mutation was associated with another mutant allele, it always resulted in severely reduced protein quantity; Figure 4) and transcriptional levels. Indeed, this mutation had been predicted by the HSF algorithm to create a new acceptor splice site (A1), which was detected in muscle mRNA by splicing-specific PCR. Furthermore, numerous cryptic splicing sites in the region surrounding this mutation obtained a high score by SSPPs analysis and were expected to cause the insertion of different portions of intron 13. Three such transcripts (A2, A3, A4) were detected in our patients by cDNA analysis. However, even though none of the algorithms used predicted the loss of the canonical acceptor splice site (and only a slight decrease in its score was obtained for two of them), we identified an aberrant transcript (WTA) carrying the insertion of the entire intron 13. All these transcripts were variably expressed and detected in the patients, but while 4 aberrant transcripts (A1, A2, A3, A4) were detected only in mutant patients, suggesting their pathogenetic effect, the variant WTA was also expressed in 10 normal controls, indicating its non-pathogenicity. We attribute the occurrence of this latter transcript to the result of an alternative splicing event that takes place in normal tissues, as previously reported [Kawabata et al., 2003; De Tullio et al., 2003].

## DISCUSSION

Despite the frequent identification of intronic changes in the *CAPN3* gene, the demonstration of their pathogenetic effect has only occasionally been sought or achieved, both due to the laborious investigations involved and, also, because mRNA/protein studies in LGMD2A are often limited by the unavailability of a muscle biopsy. Although assessment of the pathogenetic effects of such variants is challenging, the effort required is justified because successful results are crucial to offer definitive diagnoses and conclusive genetic counseling, when establishing genotype-phenotype correlations and in view of future therapies.

Using *in-silico* predictions combined with RT-PCR analyses on muscle mRNA, we demonstrated the pathological significance of 5 UVs, showing their effects on splicing and protein translation.

We showed that *CAPN3* mRNA degradation by NMD is not a drawback in muscle RT-PCR analyses, provided that these are combined with SSPPs analysis and the use of splicing-specific PCR tests. SSPPs are valuable tools to select variants that are predicted to impair correct RNA splicing, for address the subsequent variant-specific analyses at RNA level. However, they may provide contradictory or negative results, requiring the use of multiple softwares to resolve the discrepancies. Among the 4 softwares we used in this study, the most reliable and informative was the HSF, which contains also matrices for the prediction of effects on putative enhancer/silencer sequences.

Previous studies have reported different results for 3 of the 5 variants we analyzed (C.1992+1G>T, c. 1524+1G>C and c. 1746-20C>G). In particular, one study on blood mRNA identified only one aberrant transcript (D1) for the mutation C.1992+1G>T and failed to detect any mRNA for the mutation C.1524+1G>C [Blazquez et al., 2008]. We suggest that this discrepancy could be due to the different approach used. Indeed, using splicing-specific PCR, combined with *in-silico* predictions, we demonstrated that all the aberrant transcripts for the C.1992+1G>T mutation identified in muscle were expressed also in blood. Another possible explanation may be the use of a tissue other than muscle. Earlier studies reported the unreliability of RNA splicing analysis from blood samples, both because of its different splicing pattern and of the possibility that degenerated or illegitimate splicing had occurred associated with diverse storage conditions [Wimmer et al., 2000]. These observations highlight how critical the choice of the tissue to be studied is. Furthermore, because of the lack of a molecular proof, some intronic variants have been reported either as polymorphisms or as “possibly pathogenetic”, thus generating confusion and compromising a conclusive genetic counselling. This is the case of the variation c. 1746-20C>G in intron 13 [Hermanova et al., 2006; Stehlikova et al., 2007; Krahn et al., 2007; Groen et al., 2007], for which we have provided a definite demonstration of the pathogenetic effect.

In our study, the 3 mutations localized at conserved canonical donor/acceptor splice sites showed clearly their pathogenicity at both RNA and protein level, causing the loss of splice site and resulting in the absence of protein. For the 2 mutations localized in less conserved regions nearby the exon/intron boundaries, SSPPs did not predict the loss of canonical splice sites, but only provided a decreased score, indicating a higher probability of use of nearby cryptic splicing sites, which indeed we experimentally demonstrated. Patients carrying these 2 latter mutations and heterozygous for a different frame-shifting mutation showed either absent or severely reduced calpain-3 protein, indicating that if a correct splicing were still possible, it might occur to a very limited extent.

The majority of the aberrant transcripts characterized in this study both contained a frame-shifting and either were expressed at very low abundance or were detectable only by splicing-specific-PCR. This result agrees with trace amounts of the corresponding transcript identified in LGMD2A patients who were compound heterozygous for 2 null mutations [Stehlikova et al., 2007]. Conversely, the transcripts originating from the C.1524+1G>C and C.1992+1G>T variants contained in-frame insertions/deletions, which were however expressed at low abundance, possibly because alternative splicing may limit the export of mRNA, making it a target for degradation. These transcripts could potentially have generated longer/truncated protein products, which were however not detected.

One conclusion from our study is that although SSPPs analysis helps in addressing the potential effects of intronic variations, experimental demonstration of this is not always easy to do by mRNA analysis (low abundance of transcripts, NMD mechanism) and might prove unsuccessful unless the individual transcripts are identified by both splicing-specific-PCR tests and sequencing. Furthermore, SSPPs analysis proved to be very valuable and reliable in predicting aberrant splicing for variants localized in the intronic 5′ and 3′ splice-site region, whereas it was neither conclusive nor always successful in the prediction of the consequences of such loss. For this purpose, however, it was useful to check the regions surrounding the nucleotide variant for the presence of potential cryptic splicing sites and for the loss/gain of splicing regulatory elements [Faustino and Cooper, 2003; Shapiro and Senapathy, 1987].

We verified that a common effect of intronic variations in the *CAPN3* gene, occurring in all 5 mutations studied, is the aberrant splicing caused by the activation of a series of cryptic splicing sites near the mutant nucleotide, and the creation/disruption of potential silencer/enhancer motifs. Mutation analysis by SSPPs should therefore involve the sequence context, in order to obtain more informative predictions. Conversely, the occurrence of exon skipping was observed only in one case in our series, suggesting either that this a less frequent mechanism used for the variants analysed (possibly because of the presence of a nearby cryptic splice site) or that transcripts carrying exon-skipping are more unstable and prone to degradation by NMD.

Although experimental demonstration of the pathogenetic effects of intronic mutations is difficult and laborious, we found that it is often successful when this effort is based on a preliminary SSPPs analysis. This aim should be pursued more frequently because of its important consequences for clinical and genetic counselling, for establishing genotype-phenotype correlations and providing novel insights into the complex mechanism of splicing.

## Figures and Tables

**Table S1:** Software Predicted Effect of Intronic Variations at cDNA Level

		**HSF** (range 0–100)			**NetGene2** (range 0–1)			**SpliceView** (range 0–100)			**NNSplice** (range 0–1)		
					
Intronic sequence variant	Transcripts	CV for WT seq.	CV for mutant seq.	CV Var. %	CV for WT seq.	CV for mutant seq.	CV Var. %	CV for WT seq.	CV for mutant seq.	CV Var. %	CV for WT seq.	CV for mutant seq.	CV Var. %
**c.1030-1G>A**	**WTA**	79.96	51.02	−36.2 **SB**	0.19	0	**SB**	82	-	**SB**	0.69	-	**SB**
	
	**A1/ c.1030-1**	46.54	75.48	+62.2 **NS**	0	0.14	**NS**	0	79	**NS**	-	-	-

	**WTD**	86.1	59.27	−31.17 **SB**	0.93	0	**SB**	84	-	**SB**	0.91	-	**SB**
	
**c.1524+1G>C**	**D1/ c.1426**	84.76	84.76	0	-	-	-	83	83	0	-	-	-
	
	**D2/ c.1524+213**	86.43	86.43	0	-	-	-	78	78	0	-	-	-

	**WTD**	86.21	59.38	−31.13 **SB**	0.95	-	**SB**	86	-	**SB**	0.97	-	**SB**
	
**c.1992+1G>T**	**D1/ c.1992+32**	77.72	77.72	0	-	-	-	81	81	0	0.78	0.78	0
	
	*D2/ c.1943	85.38	85.38	0	-	-	-	84	84	0	-	-	-
	
	*D3/ c. 1992+96	81.2	81.2	0	-	-	-	-	-	-	-	-	-

**c.1193+6T>A**	**WTD**	94.09	91.76	−2.48	1	0.95	−5	89	86	−3.4	0.98	0.95	−3.1
	
	**D1/c.1193+32**	92.26	92.26	0	0.89	0.93	+4.5	89	89	0	0.99	0.99	0

	**WTA**	93.39	93.39	0	-	-	-	88	87	−1.14	0.95	0.93	−2.11
	
	**A1/ c.1746-21**	54.37	83.31	+53.24 **NS**	-	-	-	-	-	-	-	-	-
	
	**A2/ c.1746-88**	88.06	88.06	0	0.18	0.19	+5.55	81	81	0	0.67	0.67	0
	
**c.1746-20C>G**	**A3/ c.1746-124**	82.52	82.52	0	-	-	-	77	77	0	-	-	-
	
	**A4/ c.1746-307**	84.93	84.93	0	0.43	0.43	0	85	85	0	0.60	0.60	0
	
	*A5/ c.1746-550	86.86	86.86	0	-	-	-	80	80	0	0.65	0.65	0
	
	*A6/ c.1746-644	94.5	94.5	0	-	-	-	86	86	0	0.74	0.74	0

WT A: Wild type acceptor site; WT D: Wild type donor site; D1, D2, etc: alternative cryptic donor sites; A1, A2, etc: alternative cryptic acceptor sites; CV: consensus value; Var.%: CV variation between WT and mutated sequence; **SB**: site broken. **NS**: new site

* transcripts not detected by cDNA analysis

- site not detected by the software.

## References

[b1] Blázquez L, Azpitarte M, Sáenz A, Goicoechea M, Otaegui D, Ferrer X, Illa I, Gutierrez-Rivas E, Vilchez JJ, López de Munain A (2008). Characterization of novel CAPN3 isoforms in white blood cells: an alternative approach for limb-girdle muscular dystrophy 2A diagnosis. Neurogenetics.

[b2] Desmet FO, Hamroun D, Lalande M, Collod-Beroud G, Claustres M, Beroud C (2009). Human Splicing Finder: an online bioinformatics tool to predict splicing signals. Nucleic Acid Res.

[b3] De Tullio R, Stifanese R, Salamino F, Pontremoli S, Melloni E (2003). Characterization of a new p94-like calpain form in human lymphocytes. Biochem J.

[b4] Duno M, Sveen ML, Schwartz M, Vissing J (2008). cDNA analyses of CAPN3 enhance mutation detection and reveal a low prevalence of LGMD2A patients in Denmark. Eur J Hum Genet.

[b5] Fanin M, Nascimbeni AC, Aurino S, Tasca E, Pegoraro E, Nigro V, Angelini C (2009a). Frequency of LGMD gene mutations in Italian patients with distinct clinical phenotypes. Neurology.

[b6] Fanin M, Nascimbeni AC, Tasca E, Angelini C (2009b). How to tackle the diagnosis of limb-girdle muscular dystrophy 2A. Eur J Hum Genet.

[b7] Faustino NA, Cooper TA (2003). Pre-mRNA splicing and human disease. Genes Dev.

[b8] Groen EJ, Charlton R, Barresi R, Andreson LV, Eagle M, Hudson J, Santibanez Koref M, Straub V, Bushby KMD (2007). Analysis of the UK diagnostic strategy for limb girdle muscular dystrophy. Brain.

[b9] Haffner K, Speer A, Hubner C, Voit T, Oexle K (1998). A small in-frame deletion with in the protease domain of muscle-specific calpain-3, p94 causes early-onset lib-girdle muscular dystrophy 2A. Hum Mut.

[b10] Hermanova M, Zapletalova E, Sedlackova J, Chrobakova T, Letocha O, Kroupova I, Zamecnik J, Vondracek P, Mazanec R, Marikova T, Vohanka S, Fajkusova L (2006). Analysis of histopathologic and molecular pathological findings in Czech LGMD2A patients. Muscle Nerve.

[b11] Kawabata Y, Hata S, Ono Y, Ito Y, Suzuki K, Abe K, Sorimachi H (2003). Newly identified exons encoding novel variants of p94/calpain-3 are expressed ubiquitously and overlap the alpha-glucosidase C gene. FEBS Lett.

[b12] Krahn M, Bernard R, Pécheux C, Hammouda EH, Eymard B, Lopez de Munain A, Cobo AM, Romero N, Urtizberea A, Leturcq F, Levy N (2006). Screening of the CAPN3 gene in patients with possible LGMD2A. Clin Genet.

[b13] Krahn M, Pécheux C, Chapon F, Béroud C, Drouin-Garraud V, Laforet P, Romero NB, Penisson-Besnier I, Bernard R, Urtizberea JA, Leturcq F, Lévy N (2007). Transcriptional explorations of CAPN3 identify novel splicing mutations, a large-sized genomic deletion and evidence for messenger RNA decay. Clin Genet.

[b14] http://www.dmd.nl.

[b15] Maquat LE (2004). Nonsense-mediated mRNA decay: splicing, translation and mRNP dynamics. Nat Rev Mol Cell Biol.

[b16] Piluso G, Politano L, Aurino S, Fanin M, Ricci E, Ventriglia VM, Belsito A, Totaro A, Saccone V, Topaloglu H, Nascimbeni AC, Fulizio L, Broccolini A, Canki-Klain N, Comi LI, Nigro G, Angelini C, Nigro V (2005). Extensive scanning of the calpain-3 gene broadens the spectrum of LGMD2A phenotypes. J Med Genet.

[b17] Shapiro MB, Senapathy P (1987). RNA splice junctions of different classes of eukaryotes: sequence statistics and functional implications in gene expression. Nucleic Acid Res.

[b18] Stehlíková K, Zapletalová E, Sedlácková J, Hermanová M, Vondrácek P, Maríková T, Mazanec R, Zámecník J, Vohánka S, Fajkus J, Fajkusová L (2007). Quantitative analysis of CAPN3 transcripts in LGMD2A patients: involvement of nonsense-mediated mRNA decay. Neuromusc Disord.

[b19] Wimmer K, Eckart M, Rehder H, Fonatsch C (2000). Illegitimate splicing of the NF1 gene in healthy individuals mimics mutations-induced splicing alterations in NF1 patients. Hum Genet.

